# Towards stronger tobacco control policies to curb the smoking epidemic in Spain

**DOI:** 10.1007/s12094-024-03385-9

**Published:** 2024-02-12

**Authors:** Mónica Pérez-Ríos, Jasjit Ahluwalia, Carla Guerra-Tort, Guadalupe García, Julia Rey-Brandariz, Nerea Mourino-Castro, Ana Teijeiro, Raquel Casal-Fernández, Iñaki Galán, Leonor Varela-Lema, Alberto Ruano-Ravina

**Affiliations:** 1 Department of Preventive Medicine and Public Health, University of Santiago de Compostela, Santiago de Compostela, Spain; 2 CIBER Epidemiología y Salud Pública (CIBERESP), Madrid, Spain; 3 Health Research Institute of Santiago de Compostela (IDIS), Santiago de Compostela, Spain; 4 Department of Medicine, Alpert School of Medicine, Brown University, Providence, USA; 5 Department of Behavioral and Social Science, School of Public Health, Brown University, Providence, USA; 6 Legoretta Cancer Center, Division of Biology and Medicine, Brown University, Providence, USA; 7 National Centre for Epidemiology, Carlos III Institute of Health, Madrid, Spain; 8 Department of Preventive Medicine and Public Health, Autonomous University of Madrid/IdiPAZ, Madrid, Spain

**Keywords:** Spain, Tobacco, Smoke, Secondhand smoke, Health policy, Smoke-free policy

## Abstract

Smoking and exposure to secondhand smoke pose a significant risk to the health of populations. Although this evidence is not new, the commitment of countries to implement laws aimed at controlling consumption and eliminating exposure to secondhand smoke is uneven. Thus, in North America or in Europe, locations like California or Ireland, are pioneers in establishing policies aimed at protecting the population against smoking and secondhand smoke. Identifying measures that have worked would help control this important Public Health problem in other countries that are further behind in tobacco control policies. In Spain, there has been almost 15 years of little political action in legislation oriented to control the tobacco epidemic. If we want to achieve the tobacco endgame, new legislative measures must be implemented. In this paper, we have elucidated tobacco control policies that could be implemented and show how different countries have done so.

## Introduction

Tobacco smoking and exposure to secondhand smoke (SHS) are modifiable risk factors with a great impact on morbidity and mortality [1–3]. Smoking is an epidemic in many countries with high prevalence in certain vulnerable groups, such as minors [1, 4].

In the early 1950s, two groundbreaking reports published in the United Kingdom and the United States (U.S.) established tobacco consumption as a threat to human health [5, 6]. Since then, the efforts of governments to control the tobacco epidemic have been inconsistent, both from a geographical and a temporal perspective. Some of the earliest tobacco control initiatives were developed in the United States. For example, beginning in the 1980s, the state of California implemented tobacco control policies aimed at reducing both consumption and exposure to SHS [7]. These policies placed California as a global leader in tobacco control [8].

Another important initiative was the World Health Organization Framework Convention on Tobacco Control (WHO FCTC), which was adopted unanimously by the 56th World Health Assembly in 2003, with enforcement beginning in 2005. It was the first step in the global efforts to address the tobacco epidemic [9]. In 2008, the WHO established a technical package with the objective of assisting countries in fulfilling their commitments under the FCTC, the acronym MPOWER [10], referring to the six measures which reflects at least one provision of the WHO FCTC: M (Monitor tobacco use and prevention policies); P (Protect people from tobacco smoke); O (Offer help to quit smoking); W (Warn about the dangers of tobacco); E (Enforce bans on tobacco advertising, promotion and sponsorship); and R (Raise taxes on tobacco).

Recently, an ambitious new strategy, known as the tobacco endgame, is seeking the end of the epidemic rather than just its control [3]. With the main objective of reducing the percentage of smokers to less than 5%, it has greater possibilities of implementation in countries with a low prevalence of tobacco consumption or in those with a very rapid decline in prevalence. Different endgame initiatives, with remarkable results in combating the tobacco epidemic, have been implemented in countries such as Australia, New Zealand, or Singapore. These initiatives are worthwhile tobacco endgame strategies, since they are designed to eliminate, within a specified time frame, the structural, political, and social dynamics that sustain the tobacco epidemic [3]. Identifying the actions undertaken in different countries, and interposing them in a particular context, could help to identify which tobacco control measures are necessary to reduce prevalence.

While it is not our aim to provide a list of strategies to reduce the prevalence of tobacco smoking and exposure to secondhand smoke, this review aims to offer potential possibilities for the identification of tobacco control policies that could be implemented in Spain, relying on political will, as a comprehensive strategy to protect the population.

### Tobacco epidemiology in Spain at a glance (Box 1)

In Spain, the prevalence of tobacco consumption has been decreasing since 1987 when it was 38.4–22.1% in 2020. While the decrease in the prevalence were observed in both men and women, the decline was more marked in men (55.1% in 1987 to 25.9% in 2020 while in women it fell from 23.0 to 18.5%). Furthermore, in men, the prevalence was consistently decreasing, while in women, an increasing trend was observed until 2001 followed by a decreasing trend [11]. Regarding exposure to SHS, in 2020, 13.5% of the Spanish population aged 15 years and older was exposed in indoor places [12].

Smoking caused more than 60,000 deaths in 2018 in Spain, with 49,978 deaths in men and 12,673 in women. Mortality rates attributed to tobacco consumption show a different trend depending on sex, with a decrease in men and an increase in women [13]. Additionally, attributable mortality to SHS exposure has decreased from 2002 [14] to 2020, when SHS caused 747 deaths in those aged 35 years and older [15].

### Tobacco legislation in Spain at a glance (Box 2)

In 2005, Spain ratified the World Health Organization Framework Convention on Tobacco Control treaty (FCTC). In January 2006, Spain was one of the first European countries to implement comprehensive legislation for tobacco control, which was later modified in 2010. The law 42/2010 [16] extended the initial prohibition of smoking to all enclosed public places by including hospitality establishments, and making minimal exceptions. This law extended the smoking ban to some outdoor spaces, such as playgrounds and children's play areas, educational centers dedicated to children under 18 years of age, and the premises of health centers. As exceptions, smoking was allowed in hotel rooms, to a maximum of 30% of the total number of hotel rooms, as well as in outdoor spaces of universities and centers exclusively dedicated to adult education. Finally, prisons, medium and long-term psychiatric centers, and residences for the elderly and disabled can establish smoking outdoor areas and enable smoking closed rooms.

### Evidence-based cornerstones in tobacco control policies

#### Taxes

Spanish context: The last time Spain increased cigarette excise tax was in 2016. The tax rate for roll-your-own cigarettes and e-cigarettes is lower in contrast to manufactured cigarettes. Regarding tobacco price, in general, Spain is in the low range in comparison with other European Union countries. The Tobacco Control Scale gives Spain a low score, 12 points on a maximum of 30, with regards tobacco price [17].

Tobacco taxation is the most effective policy mechanism to reduce tobacco consumption; strengthening tobacco taxation is a key policy tool-the letter R in MPOWER denotes “Raise taxes on tobacco” [18]. The impact of price increases, through raising excise taxes, has a strong effect on the prevalence of tobacco smoking. There is a direct and inverse relationship on increasing tobacco taxes with concomitant decreasing tobacco prevalence. Two seminal works on this topic were published by Chaloupka et al. in 2010 [19] and 2012 [20]. In the 2010 paper, the evidence regarding the increase in tobacco prices and the global decrease in the prevalence of consumption in adults and young people was established as sufficient [19]. In addition, the paper also established a sufficient level of evidence relating the increase in prices with the decrease in the number of cigarettes smoked. If price increases 10%, cigarette consumption decreases by 3–5%, half of the decrease due to quitting and half to a reduction in daily consumption [21].

In 2021, the WHO technical manual on tobacco tax policy and administration [22] was published. This manual, an update of a previous one [23], set the framework for guiding governments on the development of an efficient tobacco taxation policy. It clearly establishes that the implementation of a simple, strong, and equitable tobacco excise tax structure for all tobacco products, is crucial. This excise tax structure should include regular adjustments for inflation, and income growth. The WHO technical manual also states that all tobacco products should be taxed in a comparable way. As a complement to an excise tax, pricing regulation is central to ensuring that there are no large price gaps between premium and inexpensive tobacco products, as this avoids a move to the consumption of cheaper tobacco products [22].

The tobacco lobby, aware of the effectiveness of taxation, addresses populations, governments, and policymakers with a variety of underhanded tactics. These SCARE (SCARE refers to the acronym) tactics mainly work in five arenas: Smuggling and illicit trade, Court and legal challenges, Anti-poor rhetoric, Revenue reduction, and Employment impact [22].

At the government and policymaker level, the most threatening argument is focused on revenue. This decrease could be seen as a threat by countries since the revenue that the country receives from tobacco is reduced, because of the decrease in prevalence. However, it has been observed that, for example in California, after the continued increases in taxes and the significant decreases in prevalence, the revenue collected from taxes has not decreased [21].

At the population level, the anti-poor rhetoric is the discourse. One of the tactics that the tobacco industry uses to discourage the implementation of these measures is the proposal that those smokers with fewer economic resources are more sensitive to price increases. This argument is widely dismissed, and the positive impact of tax increases has been seen in the vulnerable populations [21]. The repercussions on smuggling and illicit trade, court and legal challenges, and employment impact, has been demonstrated as low. Detailed analysis of these issues can be consulted elsewhere [20, 22].

### Smoke free outdoor areas

Spanish context: Smoking is prohibited in outdoors playgrounds and children's play areas, educational centers dedicated to children under 18 years of age, and the premises of health centers.

The evidence that there is no safe threshold of exposure to SHS has been established [24]. This evidence has made the adoption of tobacco control laws that regulate exposure to SHS common policy. However, most of these policies or regulations focus on exposure in enclosed public places, not outdoor areas.

At this juncture, the smoke-free legislation in the state of California stands out [25]. In California, cities and counties have the explicit authority to enforce restrictions in outdoor areas going beyond state laws. Although state laws mostly focus on the workplace, smoking is also prohibited within 20 feet of entryways or operable windows in public buildings, within 25 feet of outdoor playgrounds and within 250 feet of parks or facilities where a youth sporting event is taking place. Smoking is also prohibited in state parks and on state beaches except for paved roadways or parking facilities. The additional restrictions adopted by local communities include outdoor areas such as dining areas. Nevertheless, in California where smoking is prohibited in virtually all enclosed workplaces [25], the smoke-free outdoor area regulations are uneven in the different counties [26].

At this point, the child-smoke-free outdoor areas must receive attention, especially smoke-free school grounds. It is essential to establish smoke-free policies protecting outdoor school grounds and school entrances, although this is sometimes complicated due to the public nature of some of these spaces. Normalizing these outdoor smoking restrictions in schools or educational settings requires legislation. Signs should be prominently displayed at all entrances to school properties stating that all forms of tobacco use are prohibited. Implementing these policies in educational settings requires collaborative efforts. The importance of the collaboration of public health officials and teachers will be essential to increasing the adoption of the regulations [27]. We must highlight that developing smoke-free environments not only protects the population from SHS. It also denormalizes smoking, since it reduces the exposure of young people to smokers, counteracting the view that smoking is a usual adult behavior. It also assists those quitting by reducing exposure to other smokers.

Public support for outdoor smoke-free policies in places frequented by children is high, ranging from 86% for smoke-free cars with children to 76% for school grounds [28]. Despite this, smoke-free outdoor area policies are scarce worldwide. In the U.S., 8% of children live in zip codes with both smoke-free school grounds and playgrounds [29]. Differences between regions and wealth are notable, with the northeast and the wealthiest population as the most protected [29].

### Smoke free private areas

Spanish context: No legislation in relation to this cornerstone.

Smoke-free private areas are supported by the same arguments that have led to successful bans on smoking in workplaces and public settings: protecting vulnerable populations, helping smokers quit, and preventing adolescents from smoking initiation [30]. The application of regulations that protect people from SHS exposure in private places such as homes and cars is controversial since as it intrudes on one’s privacy and threatens individual rights. The dilemma, when children are involved, is whether the protection of their health overrides the right of the adult to smoke in their private spaces. Prohibition on tobacco consumption in private places where children are present is a matter of debate in many countries, however, in others, legislation has been enacted.

Regarding cars, in the U.S in 2006, Louisiana and Arkansas were the first states to prohibit tobacco consumption in private vehicles when children were present. In Louisiana, the ban focused on prohibiting smoking in cars with minors under 13 years of age, and in Arkansas, the age limit was initially established at 6 years of age, until later amendments raised it to 14. In California, the smoking ban was made effective in 2007, with an age of those under 18 years [31]. In the U.S., the prohibition of smoking in cars where children are present has been implemented unevenly, and where implemented the age limit from which it is applied is heterogeneous.

In the U.S., smoking restrictions in multi-family housing are becoming increasingly common, with governments, businesses, and property managers acting to protect residents from tobacco smoke. In 1997, Utah was the first state to pass legislation allowing landlords to ban smoking in apartments. In the first decade of the 2000s, a major property management company in Oregon implemented smoke-free policies in 8000 units across 100 properties in 5 states. Additionally, three California cities enacted ordinances prohibiting smoking in some or all units of multi-unit residential housing [30]. In 2009, the U.S. Department of Housing and Urban Development, issued a memorandum strongly encouraging public housing authorities to implement smoke-free policies in some or all of their units [30]. Despite partial implementation, some U.S. states have enacted comprehensive smoke-free policies in public multi-unit housing in 2023 [32]. Evidence suggests there is high compliance and support of smoke-free building policies among most multi-unit housing residents [33].

Other countries with smoke-free housing policies are Canada and Australia. Canada implemented the first smoke-free housing policies in 2007. The most comprehensive policies apply to multi-unit residential buildings in the Yukon Territory, Ontario, Quebec, British Columbia, and Alberta, including public housing, private rental housing, and condominiums [34, 35]. In Australia, New South Wales implemented that all new multi-unit residential buildings must be smoke free and owners of existing multi-unit residences can opt to make their buildings smoke free [35].

There is a growing recognition across Europe of the importance of smoke-free housing to protect public health, particularly children’s health. In Scotland, the public health mass media campaign “Take it Right Outside” (TiRO) reduced children's exposure to SHS in the home by 50% [36, 37]. The Scottish Government's 2018 Tobacco Control Action Plan, aims to encourage landlords to implement smoke-free home policies [38]. In the UK, the Action on Smoking and Health (ASH) recommends legislation requiring all new social housing, including balconies and gardens, to be smoke free by 2025 [39].

Another initiative that also impacts the private sphere is the non-smoking hiring policy. The Cleveland Clinic, in Ohio (U.S), was the first hospital to implement in 2007 a non-smoker hiring policy [40]. This policy remains in effect and is part of a larger initiative, “Tobacco-free environment”, with the mission to preserve and improve the health and lives of their employees, patients, and community. The aim is to create a culture of wellness that permeates the entire institution [41]. Although it was unusual for the time, it was not unique. In 2003, Weyco Inc, a medical benefits administrator, announced that it would no longer hire smokers [42]. Later in 2006, the company started testing their employees and charging a 50 U.S. dollar fee for those who failed the test and did not enroll in a smoking cessation programme [43]. Several organizations and hospitals across the U.S. have followed similar policies, some worthy of mention are: Memorial Health Care System in Tennessee, Baylor Health Care System in Texas and Franciscan Health System in Washington [41]. Despite receiving criticism upon implementation, employees and the broader community have accepted these policies [40]. In 2005, the WHO introduced hiring restrictions. The WHO stated that it “does not recruit smokers or other tobacco users who do not indicate a willingness to stop smoking” [41].

### Reducing the accessibility to tobacco products

Spanish context: Regarding the retail environment, Spain has a licensing system driven by economics. The sale of tobacco through vending machines is permitted at newsstands, in certain convenience stores, and inside hospitality venues such as hotels, bars, restaurants, dance halls, and gaming establishments. Vending machines must be in a location where they can be supervised by employees of the establishment. The minimum age for the sale and purchase of tobacco products is 18 years.

The tobacco retail environment has become a critical focus of policy interventions to help countries to achieve the tobacco endgame. Recent systematic reviews have suggested that limiting the number of tobacco retailers reduces smoking prevalence, youth initiation, and supports cessation [44, 45]. In 2011, the New Zealand government set a goal to make the country smoke free by 2025 [46]. As part of this initiative, reducing the number of tobacco retail outlets was recommended, giving local authorities the power to control their number and location [47]. Notably, in New Zealand, no license or registration is required to sell tobacco: any retail outlet is permitted to sell tobacco products, and many non-retail outlets, such as alcohol licensed premises, sports and social clubs also sell tobacco products. From July 1st 2024, a maximum of 599 tobacco retailers (354 urban, and up to 245 rural) will be legally able to sell smoked tobacco products in New Zealand [46]. The State of California requires tobacco retailers to obtain a license to sell tobacco products but there are no state laws restricting this number. However, when grading the county efforts on tobacco control, one of the areas evaluated is related to the location of the tobacco retailer. The establishment of zoning restrictions oriented to reduce the presence of tobacco retailers in locations where youth congregate is considered positive. This measure is applied in a minority of counties and is of note in Alameda County, San Francisco and Santa Cruz [26]. The promotion and selling of tobacco products in places close to colleges or high schools is one of the tactics used by the tobacco industry [48]. Licensing systems helps to reduce the number of tobacco outlets, however, when a licensing system is driven by economic rather than public health motives, it is detrimental to tobacco control efforts [49].

The sale of tobacco through vending machines is a point included in Article 16 of the WHO FCTC. This article reflects the importance of ensuring that tobacco vending machines are not accessible to minors to protect their access to tobacco products [50]. The regulation of tobacco sales through vending machines differs from country to country. Thus, countries such as Singapore, among others, have a tobacco ban sale via vending machines [51]. In the U.S, the Family Smoking Prevention and Tobacco Control Act, passed in 2009, prohibited the sale of tobacco products in vending machines, except in adults-only facilities [52].

In December 2022, New Zealand implemented the ban on tobacco sales to generations born after January 1st, 2009, preventing retailers to sell or supply smoke tobacco products to anyone born on or after that date [53, 54]. This ban will come into effect in July 2024. The same initiative was suggested in 2010 by Singapore researchers when they published a study proposing, to ban access to tobacco sales to those born since 2000 [55]. However, some difficulties related to the possibility of circumventing the ban and its consequences, prevented its implementation. Nevertheless, the Singapore government is studying how the ban is implemented in New Zealand to assess its possibilities in Singapore [56]. This difficulty led Singapore to opt for other measures such as raising the minimum age for the sale, consumption, possession and purchase of tobacco products to 21 years old [57]. The increase in the age from which tobacco can be purchased is spreading. California, in 2016, approved the law known as Tobacco 21 with great support in the general public [58]. It sets the minimum sales age at 21 years, except for the active-duty military personnel for whom the age remained at 18. This law was subsequently enacted in 2019 for the whole country [59]. Another initiative oriented to reduce sales was licensing people who smoke. In 2012, public health expert at the University of Sydney (Australia) Simon Chapman, proposed a compulsory license for smokers in the form of a smart card [60]. This card would allow verification of the identity and age (necessarily 18 or older) of the bearer, giving access to the purchase of tobacco products. The main features of the license would be an annual renewal fee, with different categories and prices depending on the intensity of consumption, a maximum daily purchase limit, and a knowledge test to qualify for the license, to ensure that new consumers make an informed choice. As an incentive, those who decided to quit would receive a refund of all the fees paid, with compound interest. Licensing would generate a registry of smokers and their purchases, with information particularly relevant for public health decision-makers [60]. The proposal soon met with objections [61, 62], and although Chapman went so far as to present the initiative to a government committee, it never moved forward.

### A foundation for tobacco control

Spanish context: In Spain, there is no formal structure that coordinates national efforts for tobacco control. At the national level, the Ministry of Health, and at the regional level, the health administration of the 17 autonomous communities and 2 autonomous cities, have the capacity to legislate tobacco control. Any law approved at the national level is enforceable throughout the country. The national surveillance systems are based on the National Health Surveys carried out in Spain approximately every 2 years. However, their sample size does not allow for representative information at the level of autonomous communities by age and sex. In addition, other limitations include the variability in its periodicity and the absence of precise questions to assess the prevalence of exposure to SHS.

To support the aforementioned cornerstones, building a foundation for tobacco control is essential. At this point, the U.S. experience could serve as a model to cement the cornerstones. In 1999, the U.S Center for Disease Control and Prevention (CDC) created the National and State Tobacco Control Program (NTCP) to coordinate national efforts to reduce tobacco-related diseases and deaths [63]. The NTCP has several goals, among them the elimination of exposure to SHS and the promotion of tobacco cessation. To respond to these goals, several strategies have been implemented; among these two crucial ones are surveillance and evaluation, and mass health communication interventions. These two related strategies are central and fundamental tools in controlling the tobacco epidemic.

Policies aimed at tobacco control must rely on an informed and educated population, public health professionals and policymakers. Furthermore, one key factor associated with success is the involvement and support of civil society that, based on informed knowledge [64], is aware of the need for laws aimed at the abolition of tobacco [65].

The availability of quality scientific information is crucial to inform society. This information, once obtained, must be disseminated as part of a health promotion and education strategy. Having up-to-date information is the basis of good risk communication. Powerful surveillance systems that monitor the prevalence of tobacco use and exposure to SHS are a valid source of information necessary for building and evaluating health messages and policies. In the U.S. among these data sources are the Behavioral Risk Factor Surveillance System, the National Health and Nutrition Examination Survey, National Health Interview Survey, the National Survey on Drug Use and Health and the National Youth Tobacco Survey. With an established periodicity and constant data collection, they are a reliable and valuable source of information. In the US, evidence-based guidelines such as the Reports of the Surgeon General on tobacco, the NCI's Tobacco Control Monograph series or the Best Practices for Comprehensive Tobacco Control Programs [66] are meant to help states build and maintain effective tobacco control programs. The Best Practices for Comprehensive Tobacco Control includes 20 objectives, such as reduce current tobacco use in adults or increase the proportion of cancer survivors; the objective’s status or the most recent data could be consulted in a friendly webpage [66].

Separating research results from political decision-makers and media is a mistake. At the political or policymaker level, knowledge translation from scientific evidence is of indisputable value [67]. The importance of having information based on, for example, high-quality systematic reviews linked to legislative changes aimed at tobacco control, is critical. However, this information should also reach the policy makers and be understandable to them [68]. We should highlight that the information derived from research studies is not easily understood by people not trained in scientific research. Adapting scientific knowledge to a lay audience is mandatory [69]. Regarding the media, they must be our trusted partner in transferring knowledge to the public to counter the influence of the tobacco industry. The evidence exists, what remains now is giving society access to it.

Smoking control legislation is not meant to stigmatize smokers. It is a measure to protect the health of the population. Countries must make the political decision to implement comprehensive tobacco control policies and avoid the influence of the tobacco industry. We must not forget that governments have the ethical responsibility to serve their population and must protect their health. Tobacco control measures should not be applied in isolation [7]. In countries like Brazil, the positive impact of the application of laws that regulate at different levels has been proven [70]. Taking all these issues into account is important if we also want to achieve the endgame, or the objective specified in the Europe’s Beating Cancer Plan, which aims for a tobacco prevalence of 5% or lower by 2040. What are we waiting for?

## Conclusion

In this paper, we have elucidated tobacco control policies that could be implemented and show how different countries have done this. We have also indicated which of these initiatives have been initiated and enforced in Spain, and it is clear that our country lags behind others in such policies. Tobacco policies driven by evidence-based new knowledge have to evolve constantly. Unfortunately, this is not the case in Spain. In Spain, there has been almost 15 years of little political action in the control of the tobacco epidemic. Again, what are we waiting for?

## Data Availability

Not applicable.

## References

[1] Dai X, Gakidou E, Lopez AD (2022). Evolution of the global smoking epidemic over the past half century: strengthening the evidence base for policy action. Tob Control.

[2] Rey-Brandariz J, Perez-Rios M, Santiago-Perez MI, Galan I, Schiaffino A, Varela-Lema L (2022). Trends in smoking-attributable mortality in Spain: 1990–2018. Eur J Public Health.

[3] Yousuf H, Hofstra M, Tijssen J, Leenen B, Lindemans JW, van Rossum A (2020). Estimated worldwide mortality attributed to secondhand tobacco smoke exposure, 1990–2016. JAMA Netw Open.

[4] Ma C, Heiland EG, Li Z, Zhao M, Liang Y, Xi B (2021). Global trends in the prevalence of secondhand smoke exposure among adolescents aged 12–16 years from 1999 to 2018: an analysis of repeated cross-sectional surveys. Lancet Glob Health.

[5] United States Department of Health, Education and Welfare. Smoking and Health. Report of the Advisory Committee on Smoking and Health to the Surgeon General of the United States Public Health Service. Washington, DC:1964. https://www.cdc.gov/tobacco/data_statistics/sgr/index.htm. Accessed 13 Nov 2023

[6] Royal College of Physicians. Smoking and health. A new Report on smoking and its effects on health from the Royal College of Physicians of London. London: 1962. https://www.rcplondon.ac.uk/projects/outputs/smoking-and-health-1962. Accessed 13 Nov 2023.

[7] Roeseler A, Burns D (2010). The quarter that changed the world. Tob Control.

[8] Cornelius M, Wang T, Jamal A, Loretan C, Willis G, Graham-Glover B (2023). State-specific prevalence of adult tobacco product use and cigarette smoking cessation behaviors, United States, 2018–2019. Prev Chronic Dis.

[9] World Health Organization (WHO). Framework convention on tobacco control. 2003. https://iris.who.int/bitstream/handle/10665/42811/9241591013.pdf?sequence=1. Accessed 25 Nov 2023

[10] World Health Organization (WHO) Report on the Global Tobacco Epidemic, (2008) The MPOWER package. Geneva, World Health Organization, 2008. Accessed 28 Oct 2023

[11] Rey-Brandariz J, Ruano-Ravina A, Guerra-Tort C, Santiago-Pérez M, Montes A, Varela-Lema L (2023). Evolución de la prevalencia de consumo de tabaco en España y sus 17 comunidades autónomas. 1987–2020. Gac Sanit.

[12] Ministerio de Sanidad. Portal Estadístico del Sistema Nacional de Salud (SNS). Base de datos de la Encuesta Nacional de Salud de España. https://www.sanidad.gob.es/estadisticas/microdatos.do . Accessed 29 Oct 2023

[13] Rey-Brandariz J, Pérez-Ríos M, Santiago-Pérez M, Galán I, Schiaffino A, Varela-Lema L (2022). Trends in smoking-attributable mortality in Spain: 1990–2018. Eur J Public Health.

[14] Lopez MJ, Perez-Rios M, Schiaffino A, Nebot M, Montes A, Ariza C (2007). Mortality attributable to passive smoking in Spain, 2002. Tob Control.

[15] Pérez-Ríos M, López-Medina DC, Guerra-Tort C, Rey-Brandariz J, Varela-Lema L, Santiago-Pérez MI (2023). Mortality attributable to environmental tobacco smoke exposure in Spain in 2020. Arch Bronconeumol.

[16] Jefatura del Estado. Ley 42/2010, de 30 de diciembre, por la que se modifica la Ley 28/2005, de 26 de diciembre, de medidas sanitarias frente al tabaquismo y reguladora de la venta, el suministro, el consumo y la publicidad de los productos del tabaco. BOE-A-2010–20138; 2010. https://www.boe.es/buscar/act.php?id=BOE-A-2010-20138 Accessed 21 Nov 2023

[17] Joossens L, Olefir L, Feliu A, Fernandez E. The Tobacco Control Scale 2021 in Europe. Brussels: Smoke Free Partnership, Catalan Institute of Oncology; 2022. https://www.tobaccocontrolscale.org/2021-edition/. Accessed 20 Nov 2023

[18] World Health Organization (WHO). Report on the global tobacco epidemic, 2023: protect people from tobacco smoke. Geneva: World Health Organization; 2023. Licence: CC BY-NC-SA 3.0 IGO

[19] Chaloupka FJ, Straif K, Leon ME, Working Group IAfRoC (2011). Effectiveness of tax and price policies in tobacco control. Tob Control.

[20] Chaloupka FJ, Yurekli A, Fong GT (2012). Tobacco taxes as a tobacco control strategy. Tob Control.

[21] Balogh EP, Dresler C, Fleury ME, Gritz ER, Kean TJ, Myers ML (2014). Reducing tobacco-related cancer incidence and mortality: summary of an institute of medicine workshop. Oncologist.

[22] WHO technical manual on tobacco tax policy and administration. Geneva: World.Health Organization; 2021. Licence: CC BY-NC-SA 3.0 IGO

[23] WHO technical manual on tobacco tax administration. Geneva: World Health Organization; 2010. License: CC BY-NC-SA 3.0 IGO.

[24] Department of Health and Human Services (US). Department of Health and Human Services, Centers for Disease Control and Prevention, Coordinating Center for Health Promotion, National Center for Chronic Disease Prevention and Health Promotion, Office on Smoking and Health. The Health Consequences of Involuntary Exposure to Tobacco Smoke: a report of the surgeon general. Atlanta (GA), 2006. https://www.ncbi.nlm.nih.gov/books/NBK44324/20669524

[25] California Labor Code. Ch 3 pt 1 div 5; 6404.5. Available at: https://leginfo.legislature.ca.gov/faces/codes_displaySection.xhtml?lawCode=LAB&sectionNum=6404.5 Accessed 26 Oct 2023

[26] American Lung Association. State of Tobacco Control 2022. California Local Grades. https://www.lung.org/. Accessed Oct 25 2023

[27] Rozema AD, Mathijssen JJ, Jansen MW, van Oers JA (2016). Schools as smoke-free zones? Barriers and facilitators to the adoption of outdoor school ground smoking bans at secondary schools. Tob Induc Dis.

[28] Boderie NW, Sheikh A, Lo E, Sheikh A, Burdorf A, van Lenthe FJ (2023). Public support for smoke-free policies in outdoor areas and (semi-)private places: a systematic review and meta-analysis. EClinicalMedicine.

[29] Lowrie C, Pearson AL, Thomson G (2018). Inequities in coverage of smokefree outdoor space policies within the United States: school grounds and playgrounds. BMC Public Health.

[30] Winickoff JP, Gottlieb M, Mello MM (2010). Regulation of smoking in public housing. N Engl J Med.

[31] California Health and Safety Code 2007. Health and Safety Code. Section 118947–118949. https://law.justia.com/codes/california/2007/hsc/118947-118949.html#:~:text=CA%20Codes%20(hsc%3A118947%2D118949)&text=118947.,which%20there%20is%20a%20minor. Accessed 26 Oct 2023

[32] American Nonsmokers’ Rights Foundation. U.S Public housing authority policies restricting or prohibiting smoking. 2023 https://no-smoke.org/wp-content/uploads/pdf/public-housing-authorities.pdf. Accessed 4 Nov 2023

[33] Snyder K, Vick JH, King BA (2016). Smoke-free multiunit housing: a review of the scientific literature. Tob Control.

[34] Kennedy RD, Ellens-Clark S, Nagge L, Douglas O, Madill C, Kaufman P (2015). A smoke-free community housing policy: changes in reported smoking behaviour-findings from Waterloo Region Canada. J Commun Health.

[35] Brooks A, Buchanan T, Oakes W (2020). Smoke-free environments: current status and remaining challenges in Australia. Public Health Res Pract.

[36] Turner S, Mackay D, Dick S, Semple S, Pell JP (2020). Associations between a smoke-free homes intervention and childhood admissions to hospital in Scotland: an interrupted time-series analysis of whole-population data. Lancet Public Health.

[37] Scottish Government. Cabinet Secretary for NHS Recovery: Health and social care. The Scottish Health Survey 2018: main report—revised 2020. 2020. https://www.gov.scot/publications/scottish-health-survey-2018-volume-1-main-report/. Accessed 5 Nov 2023

[38] Scottish Government. Population Health Directorate: Health and social care.Raising Scotland’s tobacco-free generation: our tobacco control action plan 2018. https://www.gov.scot/publications/raising-scotlands-tobacco-free-generation-tobacco-control-action-plan-2018/pages/3/ Accessed 5 Nov 2023

[39] Buckley K, Cheeseman H. Action on Smoking and Health. Smoking in the home: New solutions for a Smokefree Generation. 2018. https://ash.org.uk/uploads/FINAL-2018-Smokefree-Housing-report_exec-sum_web.pdf?v=1648034308. Accessed 5 Nov 2023

[40] McDaniel PA, Malone RE (2018). health care organizations and policy leadership: perspectives on nonsmoker-only hiring policies. Acad Med.

[41] Voigt K (2012). Ethical concerns in tobacco control nonsmoker and “nonnicotine” hiring policies: the implications of employment restrictions for tobacco control. Am J Public Health.

[42] Hospital Employee Health. Relias Media. Cleveland Clinic: New hires must be nonsmokers. 2008. https://www.reliasmedia.com/articles/13807-cleveland-clinic-new-hires-must-be-nonsmokers. Accessed 19 Oct 2023

[43] Johnson S. Smoke-free Worforce Policies. American Progress. 2006. https://www.americanprogress.org/article/smoke-free-workforce-policies. Accessed 19 Oct 2023

[44] Marsh L, Vaneckova P, Robertson L, Johnson TO, Doscher C, Raskind IG (2021). Association between density and proximity of tobacco retail outlets with smoking: a systematic review of youth studies. Health Place.

[45] Valiente R, Escobar F, Urtasun M, Franco M, Shortt NK, Sureda X (2021). Tobacco retail environment and smoking: a systematic review of geographic exposure measures and implications for future studies. Nicotine Tob Res.

[46] Thornley L, Edwards R, GT. WA. Achieiving smokefree aotearoa by 2025. 2017. https://aspire2025.files.wordpress.com/2017/08/asap-main-report-for-web2.pdf. Accessed 31 Oct 2023

[47] Marsh L, Doscher C, Robertson LA (2013). Characteristics of tobacco retailers in New Zealand. Health Place.

[48] Coughlin P, Jr. JF. A Review of R.J. Reynolds' Internal Documents Produced in Mangini vs. R.J. Reynolds Tobacco Company, Civil Number 939359 -- The Case that Rid California and the American Landscape of “Joe Camel.” 1998. https://www.industrydocuments.ucsf.edu/wp-content/uploads/2014/10/Mangini_report.pdf. Accessed 2 Nov 2023

[49] Kuipers MAG, Nuyts PAW, Willemsen MC, Kunst AE (2022). Tobacco retail licencing systems in Europe. Tob Control.

[50] World Health Organisation. WHO Framework Convention on Tobacco Control overview. 2003. https://fctc.who.int/who-fctc/overview. Accessed 15 Sep 2022

[51] Tobacco Control Laws. Legislation. 2023. https://www.tobaccocontrollaws.org/legislation. Accessed 3 Nov 2023

[52] Food and Drug Administration. Family smoking prevention and tobacco control act—an overview. 2020. https://www.fda.gov/tobacco-products/rules-regulations-and-guidance/family-smoking-prevention-and-tobacco-control-act-overview. Accessed 3 Nov 2023

[53] Smokefree Environments and Regulated Products Amendment Regulations 2023. https://www.health.govt.nz/our-work/regulation-health-and-disability-system/smoked-tobacco-products/smokefree-environments-and-regulated-products-smoked-tobacco-amendment-act. Accessed 30 Oct 2023

[54] McCall C (2022). A smoke-free generation: new Zealand’s tobacco ban. Lancet.

[55] Khoo D, Chiam Y, Ng P, Berrick AJ, Koong HN (2010). Phasing-out tobacco: proposal to deny access to tobacco for those born from 2000. Tob Control.

[56] Ministry of Health Singapore. Tobacco-free generation. 2023. https://www.moh.gov.sg/news-highlights/details/tobacco-free-generation. Accessed 19 Oct 2023

[57] Amul GGH, Ong SE, Mohd Khalib A, Yoong JS (2022). Time for tobacco-free generations in the Western Pacific?. Lancet Reg Health West Pac.

[58] Zhang X, Vuong TD, Andersen-Rodgers E, Roeseler A (2018). Evaluation of California's 'Tobacco 21' law. Tob Control.

[59] Tobacco 21. 2021. https://www.fda.gov/tobacco-products/retail-sales-tobacco-products/tobacco-2. Accessed 14 Nov 2023

[60] Chapman S (2012). The case for a smoker’s license. PLoS Med.

[61] Collin J (2012). The case against a smoker’s license. PLoS Med.

[62] Magnusson RS, Currow DC (2013). Could a scheme for licensing smokers work in Australia?. Med J Aust.

[63] Centers for Disease Control and Prevention. 2021. https://www.cdc.gov/tobacco/stateandcommunity/tobacco-control/index.htm. Accessed 15 Oct 2023

[64] Malone RE (2016). The race to a tobacco endgame. Tob Control.

[65] Malone RE, Proctor RN (2022). Prohibition no, abolition yes! Rethinking how we talk about ending the cigarette epidemic. Tob Control.

[66] National Center for Chronic Disease Prevention and Health Promotion, Office on Smoking and Health. Best practices for comprehensive tobacco control programs. Preventive Services Task Force. 2014. https://www.cdc.gov/tobacco/stateandcommunity/guides/pdfs/2014/comprehensive.pdf. Accessed 2 Nov 2023

[67] Lavis JN, Posada FB, Haines A, Osei E (2004). Use of research to inform public policymaking. Lancet.

[68] Pope C, Mays N, Popay J (2006). Informing policy making and management in healthcare: the place for synthesis. Healthc Policy.

[69] Happer C, Philo G (2013). The role of the media in the construction of public belief and social change. J Soc Pol Psycol.

[70] Portes LH, Machado CV, Turci SRB (2018). History of Brazil’s tobacco control policy from 1986 to 2016. Cad Saude Publica.

